# High-density lipoproteins mediate small RNA intercellular communication between dendritic cells and macrophages

**DOI:** 10.1016/j.jlr.2023.100328

**Published:** 2023-01-07

**Authors:** Mark Castleberry, Chase A. Raby, Anca Ifrim, Yasuhiro Shibata, Sachi Matsushita, Shinya Ugawa, Yutaka Miura, Atsushi Hori, Takashi Miida, MacRae F. Linton, Danielle L. Michell, Maki Tsujita, Kasey C. Vickers

**Affiliations:** 1 Department of Medicine, Vanderbilt University Medical Center, Nashville, TN, USA; 2 Department of Anatomy and Neuroscience, Nagoya City University Graduate School of Medical Sciences, Nagoya, Aichi, Japan; 3 Department of Biochemistry, School of Dentistry, Aichi Gakuin University, Nagoya, Aichi, Japan; 4 Department of Nutrition, Shigakkan University, Obu, Aichi, Japan; 5 Department of Clinical Laboratory Medicine, Juntendo University Faculty of Medicine, Tokyo, Japan; 6 Department of Biochemistry, Nagoya City University Graduate School of Medical Sciences, Nagoya, Japan

**Keywords:** apolipoproteins, extracellular RNA, intercellular communication, lipoproteins, macrophage, BLT-1, Block lipid transport-1, BMDC, bone marrow-derived dendritic cell, BMDM, bone marrow-derived macrophage, DGUC, density-gradient ultracentrifugation, EV, extracellular vesicles, FH, familial hypercholesterolemia, miRNA, microRNA, MST, microscale thermophoresis, qPCR, quantitative PCR, SEC, size-exclusion chromatography, SR-BI, Scavenger receptor, class B type I, sRNA, small RNAs, TG, triglyceride, TLR, toll-like receptor

## Abstract

HDL are dynamic transporters of diverse molecular cargo and play critical roles in lipid metabolism and inflammation. We have previously reported that HDL transport both host and nonhost small RNAs (sRNA) based on quantitative PCR and sRNA sequencing approaches; however, these methods require RNA isolation steps which have potential biases and may not isolate certain forms of RNA molecules from samples. HDL have also been reported to accept functional sRNAs from donor macrophages and deliver them to recipient endothelial cells; however, using PCR to trace HDL-sRNA intercellular communication has major limitations. The present study aims to overcome these technical barriers and further understand the pathways involved in HDL-mediated bidirectional flux of sRNAs between immune cells. To overcome these technical limitations, SYTO RNASelect, a lipid-penetrating RNA dye, was used to quantify a) overall HDL-sRNA content, b) bidirectional flux of sRNAs between HDL and immune cells, c) HDL-mediated intercellular communication between immune cells, and d) HDL-mediated RNA export changes in disease. Live cell imaging and loss-of-function assays indicate that the endo-lysosomal system plays a critical role in macrophage storage and export of HDL-sRNAs. These results identify HDL as a substantive mediator of intercellular communication between immune cells and demonstrate the importance of endocytosis for recipient cells of HDL-sRNAs. Utilizing a lipid-penetrating RNA-specific fluorescence dye, we were able to both quantify the absolute concentration of sRNAs transported by HDL and characterize HDL-mediated intercellular RNA transport between immune cells.

Nearly a century has passed since the discovery of HDL and most of HDL research has been focused on intercellular transport of lipids (e.g., cholesterol, phospholipids) (1, 2). Although there is convincing evidence of an inverse relationship between HDL-C levels and risk of coronary artery disease, drug trials that have attempted to raise HDL-C levels for therapeutic benefit, and have either failed to lower incidence of coronary artery disease or have increased cardiovascular events (2, 3). Since the failures of these HDL-C clinical trials, the HDL field began to look towards HDL quality, function, and cargo as opposed to simply HDL-C levels. Cholesterol is but one of many classes of cargo circulating on HDL particles, as HDL also transport small RNAs (sRNAs), various proteins, and diverse metabolites. Recent HDL studies have been designed to investigate HDL cargo and quality in terms of functional integrity and risk of cardiovascular events (2, 4, 5).

The initial discovery that HDL transport functional microRNAs (miRNA) has led to further profiling studies and the identification of multiple other classes of sRNAs on HDL (6, 7, 8). For example, other classes of host-derived sRNAs have been found, including sRNAs-derived from parent transcripts of tRNAs, rRNAs, and small nucleolar RNAs (9). Moreover, nonhost sRNAs originating from bacteria, fungi, and viruses in our microbiome, diet, and environment have also been detected on circulating lipoproteins (9, 10). HDL-sRNAs generally range from 10 to 60 nucleotides in length (8).

Multiple techniques have been utilized to quantify HDL-sRNAs and evaluate their acquisition from and delivery to cells. Based on quantitative PCR (qPCR), we have previously shown that HDL, complexed with exogenous sRNAs, have the capacity to deliver sRNAs to recipient hepatocytes (6). On the contrary, reconstituted HDL have been reported to accept miRNA efflux from J774 macrophages transfected with exogenous sRNAs in vitro (6). Despite technological advances in sRNA sequencing and qPCR, which have increased our ability to identify and characterize various HDL-sRNAs, these methods require RNA isolation prior to the generation of cDNA and downstream analyses (9, 10, 11). These processing steps can to lead to sample loss and may not isolate all sRNAs, thus leading to an incomplete view of HDL-mediated RNA trafficking (10, 11). As the processes that are involved in the acquisition, transfer, and delivery of HDL-sRNAs are poorly understood, the technical limitations posed by the current methodologies of evaluating sRNAs are impeding our understanding of these basic mechanisms. Thus, it is critical to overcome these impediments by evaluating sRNA mobilization through methodologies that allow for absolute quantification and do not require the use of techniques that may lead to an incomplete sampling of sRNAs. SYTO RNASelect appears to overcome these barriers and facilitates meaningful observations. We hypothesize that HDL mediates the bidirectional flux of sRNAs from macrophage endosomes. Utilizing a lipid-penetrating RNA-specific fluorescence dye, we were able to both quantify the absolute concentration of sRNAs transported by HDL and characterize HDL-mediated intercellular RNA transport between immune cells.

## MATERIALS AND METHODS

### Human sample population

The study population included N = 25 healthy adult patients (n = 18 females, n = 7 males). Blood samples were collected following informed written consent from patients and experiments were performed under institutional review board approved protocols from Vanderbilt University Medical Center. These studies abide by the Declaration of Helsinki principles.

### Isolation of lipoproteins by density-gradient ultracentrifugation

Blood was collected in sodium citrate-containing tubes and plasma was isolated utilizing centrifugation at 1,500 *g* for 15 min. Plasma was then frozen and shipped on dry ice to Vanderbilt University Medical Center for processing. Plasma was thawed and filtered through a 0.45 μm syringe filter and its density was adjusted to 1.025 g/ml with KBr. The sample was overlayed with 1.019 g/ml saline buffer, centrifuged at 40,000 R.P.M. (Beckman Ultracentrifuge, SW-40Ti rotor) for 24 h at 4°C, and the resultant superior layer (i.e.,VLDL) was isolated. The density of the remaining sample was adjusted to 1.080 g/ml with KBr, overlayed with 1.063 g/ml saline, centrifuged, and the resultant superior layer (i.e., LDL) was collected. The density of the remaining sample was adjusted to 1.225 g/ml with KBr, overlayed with 1.210 g/ml saline, centrifuged, and the resultant superior layer (i.e., HDL) was collected. Lipoprotein samples were dialyzed into PBS buffer (Thermo Scientific, BP399) for four exchanges into 4 L buffer utilizing dialysis tubing (Thermo Scientific, 68,100).

### Protein and lipid quantification

Protein quantification assays were performed by BCA protein assay kit (Pierce, 23,225). Total cholesterol quantification assays were performed utilizing cholesterol reagent (Pointe Scientific, C7510) and a cholesterol standard (C7509). Triglyceride (TG) quantification assays were performed utilizing TG reagent (Pointe Scientific, T7532) and a glycerol standard (G7793). Phospholipid C quantification assays were performed utilizing phospholipid C kit (FUJIFILM Medical Systems USA, NC9993780). Assays were performed in a 96-well format according to the manufacturer’s protocol, quantified by a BioTek Synergy Mx microplate reader, and analyzed using the Gen5 software (version 2.01.14).

### SYTO fluorescence quantification of RNA

RNA concentration was quantified utilizing an RNA-specific fluorescent dye (Sigma, S32703) and standard curve prepared by serial dilutions of a purified oligonucleotide (5′-rArGrArGrArArCrUrCrGrGrGrUrGrArArGrGrArArCrU-3′; Integrated DNA Technologies). Replicates of standards and samples were loaded into a 384-well, 96-well, or 24-well fluorescence plate and background fluorescence was measured (490/530 nm). The dye solution (50 μM) was added to each well to a final concentration of 10 μM and incubated at 37°C for 25 min. The fluorescence of each sample was then measured (490/530 nm), and the background fluorescence was subtracted. The concentrations of the samples were quantified utilizing the single-stranded sRNA standard curve.

### Microscale thermophoresis

Fluorescence-labelled tRNA-derived sRNA (tDR-GlyGCC: GCAUUGGUGGUUCAGUGGUAGAAUUCUCGC/3AlexF647N, 100 nM, IDT) was denatured and incubated (1:1) with serial dilutions of density-gradient ultracentrifugation (DGUC)-VLDL, DGUC-LDL, or DGUC-HDL in PBS for 5 min and then loaded in glass capillaries (NanoTemper Technologies) and tested on the Monolith NT.115 instrument (NanoTemper Technologies) as previously described (12). Microscale thermophoresis (MST) traces were acquired using the MO.Control software (v1.6, NanoTemper Technologies) and normalized binding curves from repeated measurements were analyzed using the MO.Affinity Analysis software (v2.3, NanoTemper Technologies). Binding affinity was determined by EC_50_.

### RNA isolation

Cellular RNA from J774 macrophages were purified using the Total RNA Purification Kit (Norgen, 17,200) according to the manufacturer’s protocol. RNA isolated from lipoproteins, purified by DGUC, was purified by RNeasy Mini Kit (Qiagen, 74,104) according to the manufacturer’s protocol.

### Spectrophotometry

Assays were performed using a Take3 Micro-Volume Pate (Biotek) according to the manufacturer’s protocol, quantified with a BioTek Synergy Mx microplate reader, and analyzed using the Gen5 software (version 2.01.14).

### Electrophoretic mobility assays

Assays were performed using the Agilent RNA 6,000 Pico Kit (Agilent, 5067-1513) and processed using a 2,100 Bioanalyzer instrument (Agilent G2939BA), according to the manufacturer’s protocol.

### RNA digestion

RNA digestion was performed by RNase I treatments (Thermo Scientific, EN0601), according to the manufacturer's protocol. DNA digestion was performed with dsDNase (Thermo Scientific, EN0771), according to the manufacturer's protocol.

### Size-exclusion chromatography

Samples were filtered through a 0.2 μm polyvinylidene difluoride centrifugal spin filter (Millipore, UFC30GV0S,) and were brought to a 1 ml total volume with PBS prior to injection into the sample loop of the ÄKTA™ Pure chromatography system (Cytiva). Samples were fractionated utilizing three tandem Superdex® 200 Increase 10/300 Gl columns (Cytiva, GE28-9909-44) in a Tris-HCl buffer (10 mM Tris-HCl, 150 mM NaCl, 0.2% NaN_3_, and pH 7.4). The sample loop was emptied with 5 ml of running buffer prior to a 91.5 ml fractionation period (0.3 ml/min) where 1.5 ml fractions were collected in 96-well plates (Eppendorf, E951033600).

### Animal studies

C57BL/6J wildtype mice were bred from mice obtained from Jackson Laboratory under active protocols approved by the Vanderbilt Institutional Animal Care and Usage Committee. Mice were housed with a 12h light/dark cycle with standard chow diet (NIH-31) and water ad libitum. Male and female mice ages 8–16 weeks old were used in this study. In all animal experiments described in this study, mice were euthanized by inhalation of 5% isoflurane utilizing the drop jar dosing method and cervical dislocation was performed to ensure the death of the animal.

### Tissue culture

J774A.1 cells (ATCC) were purchased and cultured according to the manufacturer’s protocols. Macrophages were labeled with SYTO RNASelect Green Fluorescent cell stain according to the manufacturer’s protocol. In experiments with HDL treatments, DGUC HDL were added in media to achieve a final protein concentration of 1 mg/ml in DMEM, Thermo Fisher supplemented with 1% penicillin/streptomycin (Gibco), L-glutamine (Thermo Fisher, 300 mg/L), and sodium pyruvate (110 mg/L). DGUC LDL and transferrin were used as controls for the intercellular RNA transfer assay at final concentration of 0.5 mg/ml in DMEM media. Cell treatments were incubated for 24 h, except RNA efflux experiments, including those with small molecule inhibitors, which were incubated for 4 h. The small molecule inhibitors Block lipid transport-1 (BLT-1, Sigma-Aldrich) and Dynasore (Sigma-Aldrich) were added to media at 10 μM final concentration, 1 h prior to and during RNA export. Scavenger receptor, class B type I (SR-BI) blocking antibodies (Novus Biologicals, NB400-113) were added to the media 1 h prior to and during RNA export experiments at a 1:500 dilution in media, according to the manufacturer’s protocol. Media were isolated from cells and centrifuged at 500 *g* in a swing bucket rotor for 15 min prior to analyses. Bone marrow cells were isolated from mouse femurs and tibiae, and cultured in treated-cell culture plates for seven days in DMEM supplemented with 10% heat-inactivated fetal bovine serum (Thermo Fisher), 1% penicillin/streptomycin (Gibco), L-glutamine (300 mg/L, Thermo Fisher), sodium pyruvate (110 mg/L), and murine cytokines GM-CSF (40 ng/ml) or GM-CSF (20 ng/ml) and IL-4 (5 ng/ml) for differentiation into bone marrow derived macrophages or dendritic cells (BMDMs or BMDCs), as previously described (13). RNA labeling and HDL treatments were performed as previously outlined above.

### Confocal microscopy

Macrophages were plated in uncoated 35 mm dishes (Part#: P35G-1.5-14-C, Mattek) at a cell density of 25,000 cells/ml media and grown to approximately 70% confluency prior to imaging. Live cell imaging was performed at 63x resolution using a Zeiss LSM 880 microscope, and analyses were completed with Zen Black software (Zeiss, Zen 2.1 SP1), and samples were maintained at 37°C and at 5% CO_2_ throughout imaging. Image panels were prepared with ImageJ software (Version 1.53e) and scale bars lengths represent a 20 nm. Unless specifically outlined, Hoechst 33,342 (Invitrogen, H3570), LysoTracker Deep Red (Invitrogen, L12492), and SYTO RNASelect Green Fluorescent cell Stain (Invitrogen, S32703) were used for live cell imaging in accordance with the manufacturer’s protocol.

### Statistical analyses

When comparing between two independent groups, unpaired two-tailed *t*-tests were applied. When comparing the means of multiple groups, One-Way ANOVA analyses were performed with Tukey’s post-test to correct for multiple comparisons. To display sample dispersion, values were reported as either the sample mean ± standard deviations or violin plots showing median and quartile ranges. A *P*-values <0.05 were considered statistically significant. Graph generation and statistical analyses were performed utilizing the Graphpad Prism 9 software.

## RESULTS

### Quantification of total lipoprotein RNA levels

**Fig. 1:**
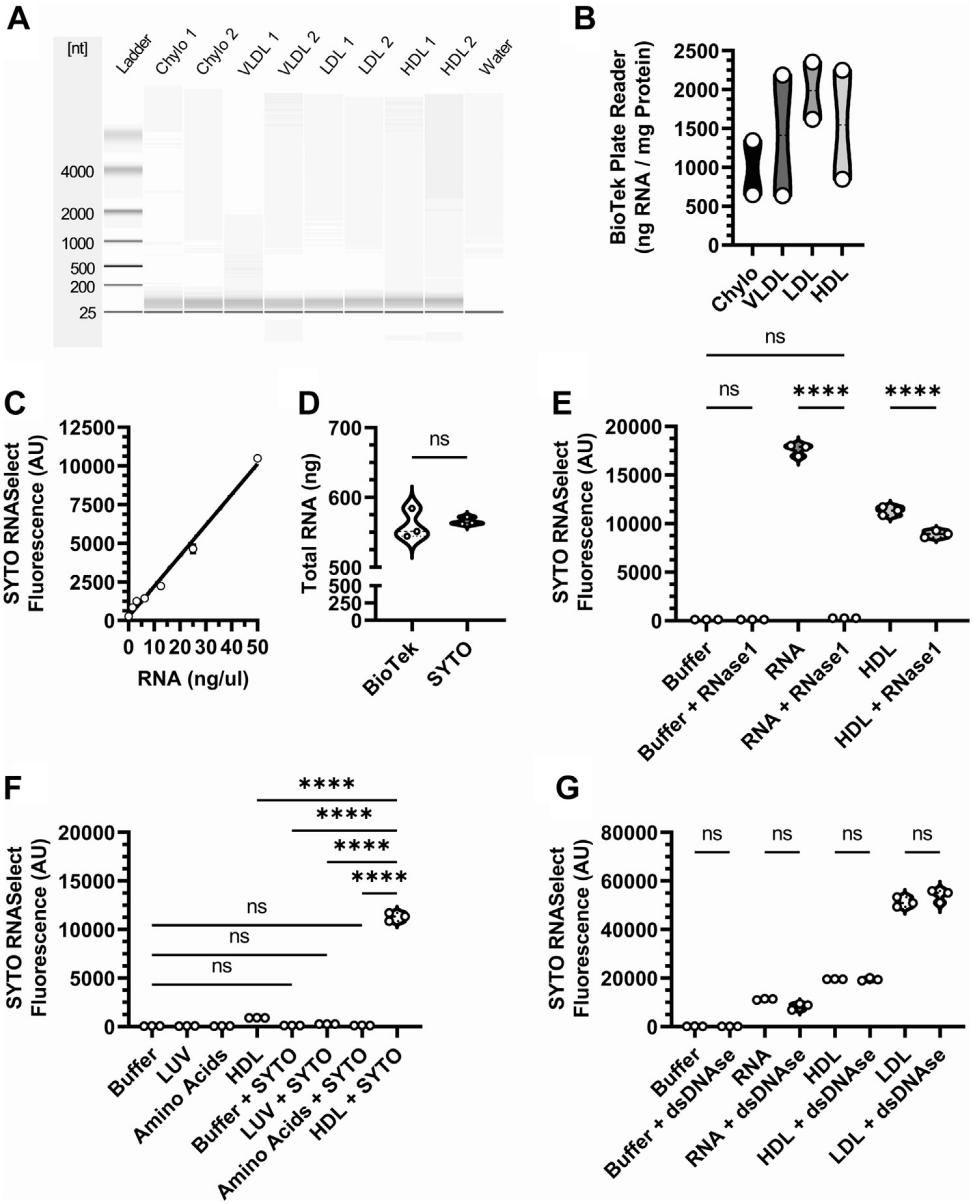
Quantification and characterization of total lipoprotein RNA. A: Isolated RNA samples from purified lipoproteins were characterized by bioanalyzer electrophoretic mobility assays through a multichannel chip and compared to purified standards. B: The yields of RNA isolated from lipoprotein samples were calculated utilizing UV spectroscopy and normalized to total protein (n = 2 per group). C: RNA dye (SYTO Green RNASelect) fluorescence was analyzed by a plate reader on a standard curve that was generated through serial dilutions of a synthesized single stranded RNA oligomer (n = 3 per group). D: Total cellular RNA was quantified utilizing UV spectroscopy and by fluorescence (n = 3 per group). Fluorescence was quantified in samples containing purified RNA (E, F) and HDL (E, F), after treatment with either RNase1 (E) or dsDNAse1 (F) (n = 3 per group). The RNA fluorescence of independent HDL (G) samples were compared to buffer, large unilamellar vesicles, and a complex of purified amino acids (n=3 per group). Values are reported as either mean ± SD (C) or violin plots showing median, quartile ranges, and all individual values (B, D, E, F, G). Student’s *t-*test (D) and one-way ANOVA (Tukey test) (E, F, G) results were as follows: ns *P* ≥ 0.05 or ∗∗∗∗ *P* < 0.0001.

The most common methods of assessing extracellular sRNA levels in biofluids involve RNA isolation steps, including solvents and silica frit-based columns, which likely have inherent biases. To quantify total RNA levels in isolated RNA samples, spectrophotometry and/or electrophoresis approaches are normally completed; however, these are time-consuming and lack sensitivity. Here, chloroform and ethanol extraction of RNA with silica frit-containing columns were used to isolate RNA from human lipoproteins, and total RNA content was assessed by bioanalyzer analysis (Agilent) (Fig. 1A). These electrophoretic mobility assays were able to detect total RNA in all lipoprotein samples (500-2,500 ng RNA / mg total protein) and provide information on RNA length on lipoproteins (Fig. 1A) by comparing the lipoprotein-derived sRNAs to RNA standards found in the assays reference ladder. By Agilent bioanalyzer, HDL were found to contain 4.3–6.7 ng RNA / mg HDL total protein (Fig. 1A). Spectrophotometry analyses of lipoprotein sRNAs were completed using the Take3 microplate reader, and lipoproteins were observed to contain 500-2,500 ng RNA / mg total protein. By this method, HDL were found to harbor 845–2,247 ng RNA / mg HDL total protein (Fig. 1B). To quantify candidate sRNAs in isolated RNA samples, qPCR or droplet digital PCR can be used after reverse transcription and cDNA synthesis. The barrier to assessing the total levels of sRNAs on extracellular carriers, including lipoproteins, is the lack of ability to quantify total sRNA levels without RNA isolation, cDNA conversion, or spectrophotometric steps. Here, we sought to use SYTO RNA Select for isolation-free absolute quantification of HDL-sRNAs.

To validate our isolation-free SYTO approach, we sought to analyze the congruency between a standard quantification technique and our method to quantify the concentration of isolated RNA. To quantify total RNA levels utilizing SYTO, we first prepared and tested a sRNA standard curve using serial dilutions of a synthesized single-stranded RNA oligoribonucleotide that resulted in a linear function which could be used for quantification (Fig. 1C). When comparing the SYTO-based standard curve quantification method with spectrophotometric quantification, the two methods were identical and showed no statistical difference (*P* > 0.1) between their calculations of isolated macrophage RNA (Fig. 1D). We then aimed to characterize our ability to use SYTO to quantify RNA from complex biological samples without first isolating RNA. The SYTO probe only emits fluorescence when it is bound to RNA and is sensitive to RNA degradation, as indicated by a reduction in fluorescence signal after treatment of RNA with RNase 1 (Figs. 1E and S1A). The treatment of purified cellular RNA with RNase 1 reduced SYTO fluorescence to levels that were not significantly different from control PBS buffer levels, which contained no RNA (Fig. 1E). However, RNase 1 treatment of DGUC-purified HDL and LDL did not eliminate SYTO fluorescence, suggesting that lipoproteins confer some protection of sRNA cargo against RNase digestion (Figs. 1E and S1A). Of note, we did observe a small but significant reduction in HDL total RNA levels with RNase 1 treatments (Fig. 1E). Unlike the results from the RNase1 digestion study, there were no observed decreases in SYTO fluorescence after treatment of purified RNA, HDL, or LDL with dsDNAse 1 (Fig. 1F). To demonstrate that SYTO only stains RNA on HDL and does not react with other HDL components, SYTO signal was measured in samples containing HDL components, including cholesterol, TG, PC, and amino acids (Fig. 1G). Unlike both HDL and LDL, the SYTO fluorescence signal of large unilamellar vesicles, loaded by equal PC, failed to show an increase in signal above the background of untreated PBS (Figs. 1G and S1B). Likewise, there were no observed significant differences in SYTO signal between a SYTO-treated mixture of purified amino acids, loaded by equal concentrations to HDL, and untreated PBS buffer (Fig. 1G). These results demonstrate that the absolute concentration of HDL-RNA can be accurately quantified with a specific RNA dye (SYTO) without the need for RNA isolation or physical disruption of complex biological samples (i.e., lipoproteins).

### Differentiation of lipoprotein RNA cargo

**Fig. 2:**
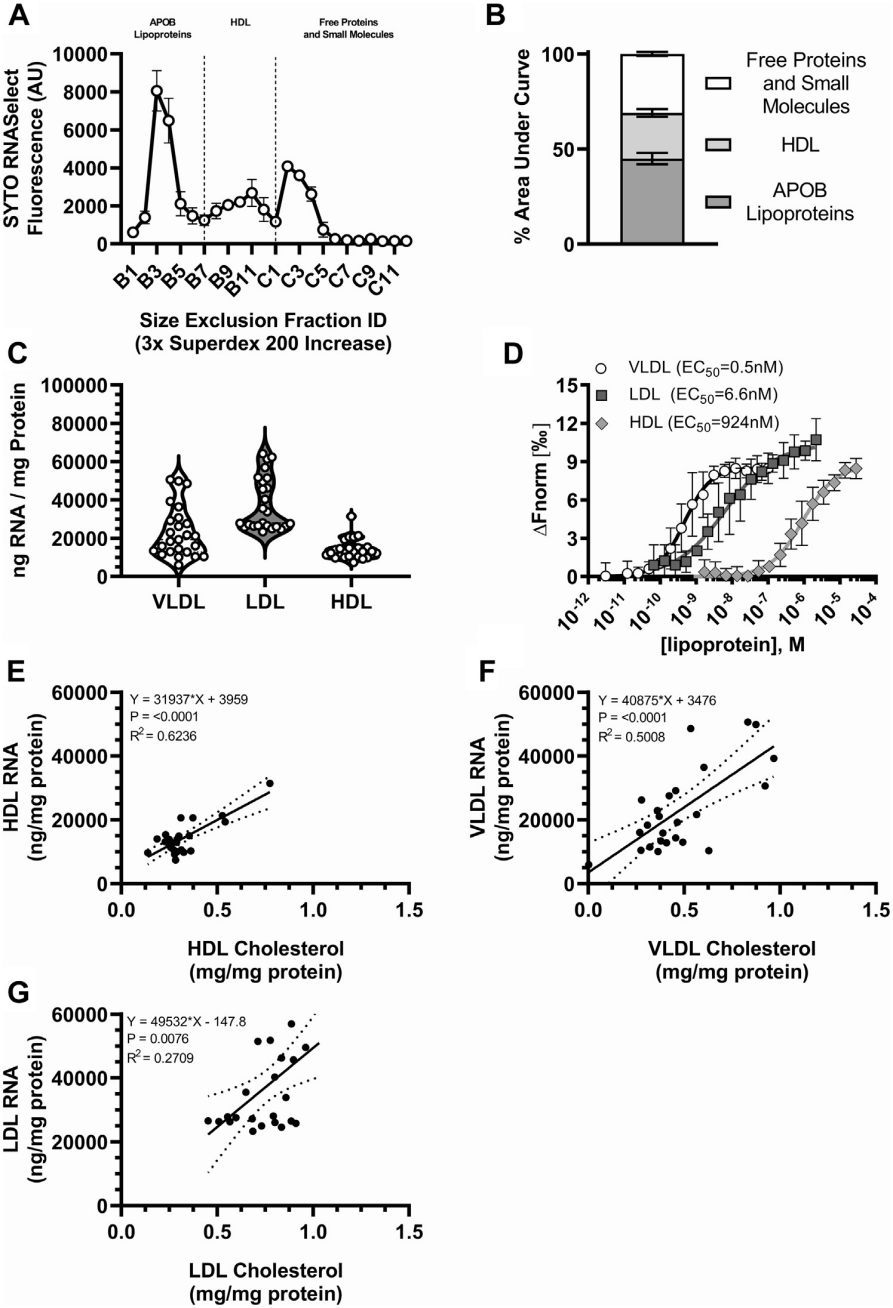
Differentiation of lipoprotein RNA cargo. Human lipoprotein RNs, utilizing plasma and DGUC isolated lipoproteins, were analyzed utilizing fluorescence. A: Chromatogram of RNA fluorescence across FPLC fractions (n = 3) from human plasma. B: Area under curve analysis of RNA chromatogram (n = 3). C: The RNA cargo of DGUC lipoproteins samples was quantified utilizing fluorescence and normalized to total protein of each sample (n = 25 per group). D: Binding affinities (EC_50_) of DGUC lipoproteins to fluorescently labeled small RNAs were determined using microscale thermophoresis (n = 3 per group). Total cholesterol concentrations were quantified by colorimetric analyses and correlated to their RNA cargo (n = 25 per group) in HDL (E), VLDL (F), and LDL (G). Values are reported as either mean ± SD (A, B, D), violin plots showing median, quartile ranges, and all individual values (C), or linear regression plots showing all individual values (E, F, G). Regression results were plotted with the best line of fit and 95% confidence bands. FPLC, fast protein liquid chromatography; DGUC density-gradient ultracentrifugation.

Extracellular RNA are transported and protected from degradation in plasma by numerous lipid- and protein-based carriers, including lipoproteins, extracellular vesicles (EV), exomeres, and supermeres (6, 14, 15). To assess the distribution of extracellular RNA across small carriers (<200 nm in diameter), human plasma samples were filtered (0.20 μm) and fractionated by size-exclusion chromatography (SEC). The distribution of total RNA was analyzed by SYTO fluorescence in each fraction (Fig. 2A). Fractions corresponding to APOB-containing lipoproteins accounted for 45% of total observed RNA signal, as reported by SYTO fluorescence. Fractions representing HDL accounted for 24%, and fractions containing most free proteins accounted for 31% overall extracellular RNA content in carriers <220 nm in diameter (Fig. 2A, B). To quantify total RNA on HDL and other lipoproteins without RNA isolation, DGUC was used to purify lipoproteins from human plasma for SYTO analyses. Based on a standard curve of single-stranded oligoribonucleotide, VLDL particles were found to contain 23,008.3 ng RNA / mg total protein and LDL particles were calculated to harbor 37,602.2 ng RNA / mg total protein. HDL particles were found to transport 14,138.5 ng RNA/ mg total protein (Fig. 2C). No significant differences between total HDL RNA cargo were observed between HDL purified by SEC, followed by Cleanascite purification, and HDL isolated by DGUC (supplemental Fig. S2). These results suggest that APOB-containing lipoproteins may contain more total RNA than HDL and free proteins (e.g., ribonucleoprotein complexes). These findings may suggest differential binding affinity towards sRNAs among lipoprotein classes. To differentiate lipoprotein binding affinities to extracellular sRNA, MST assays were used. Due to the large molecular mass of VLDL, binding affinities were calculated as EC_50_. Lipoproteins were isolated by DGUC and incubated with a fluorescence-labeled tDR-GlyGCC, a 30 nucleotide sRNA derived from parent tRNA GlyGCC that is highly-abundant on lipoproteins (9). Results from MST showed that APOB-containing lipoproteins likely have higher affinity towards extracellular sRNAs than HDL (Fig. 2D). These results agree with the observed increase in total RNA content detected by SYTO on APOB-containing lipoproteins compared to HDL; however, HDL showed strong-to-moderate affinity to tDR-GlyGCC as well (Fig. 2D). To assess the relationship between lipoprotein RNA content and lipid cargo, colorimetric analyses were performed to quantify the major lipid components of lipoproteins (Figs. 2E–G and S3). A significant correlation (*P* < 0.0001, *R*^2^ = 0.623) was observed between the total cholesterol content on HDL with its RNA cargo (Fig. 2E). This correlation was also observed with VLDL (*P* < 0.0001, *R*^2^ = 0.5) and LDL (*P* < 0.01, *R*^2^ = 0.271) (Fig. 2F, G). There were no statistically significant correlations between either PC or TG with HDL-RNA cargo (supplemental Fig. S3). There was a significant correlation (*P* < 0.01, *R*^2^ = 0.319) observed between PC and RNA in VLDL (supplemental Fig. S3) and a significant correlation (*P* < 0.01, *R*^2^ = 0.258) between TG and RNA in LDL (supplemental Fig. S3). Results from these studies support that HDL, along with APOB-containing lipoproteins, transport μg quantities of RNA per mg of total protein.

**Fig. 3:**
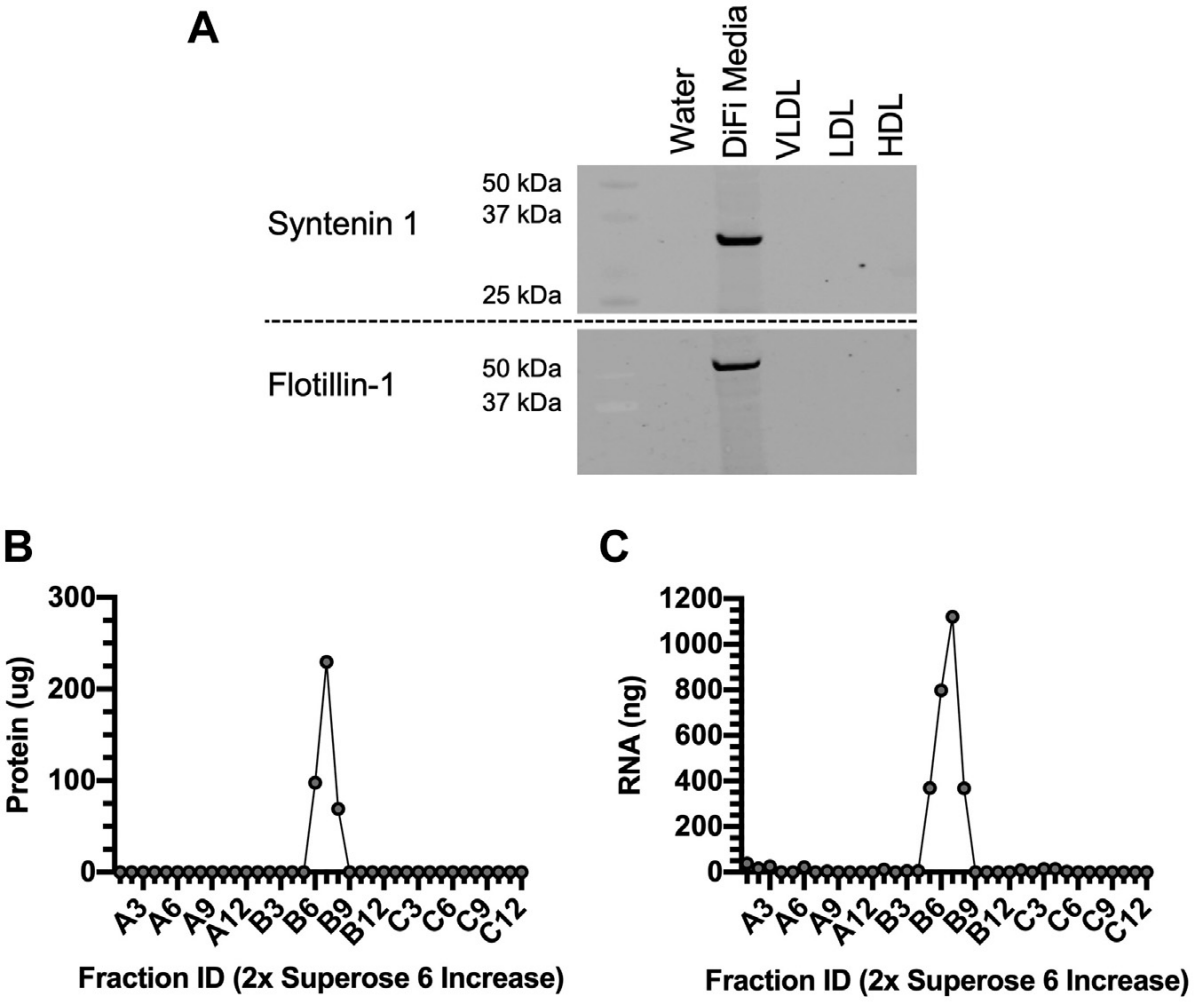
Characterization of the purity of isolated lipoproteins. A: Lipoproteins (i.e., VLDL, LDL, HDL) purified from plasma using density-gradient ultracentrifugation and were analyzed by western blotting for the presence of Syntenin-1 and Flotillin-1. Purified HDL were fractionated using size-exclusion chromatography and the total protein (B) and RNA (C) values were quantified in the eluent fractions (n = 1 per group).

Some classes of EVs have been reported to copurify with lipoproteins when using density-based isolation methods. Therefore, we sought to assess potential EV contamination in our DGUC-purified lipoprotein samples (16). We first tested our samples for EV protein markers by western blotting (Fig. 3A) (17). While we were able to detect the presence of EV markers Syntenin-1 and Flotillin-1 in our positive control sample, concentrated media from colorectal cancer cells (DiFi), we were unable to detect these proteins in our DGUC-purified lipoproteins (i.e., VLDL, LDL, HDL) (Fig. 3A) (14). Although EVs have a far greater diameter than HDL, it has been shown that the density range of EVs almost entirely overlap with HDL. Thus, we used SEC to access potential contamination of EVs within our DGUC-purified HDL by fractionating our samples by size and quantifying total protein and RNA within the eluant nonHDL fractions (Fig. 3B, C). These analyses indicate that there are no detectable levels of protein or RNA above the baseline in our assays within the size-range of EVs; however, we see robust signal in both protein and RNA that corresponds to the distinct and smaller size range of HDL (Fig. 3B, C). With these results in mind, there is no evidence to support the presence of contaminating EVs within our DGUC-purified lipoproteins. Although EVs may be present in our samples at concentrations well below our threshold of detection, these extremely minor contaminants likely do not bias or alter our assays, results, or conclusions.

### HDL acceptance of cellular RNA from macrophages

**Fig. 4:**
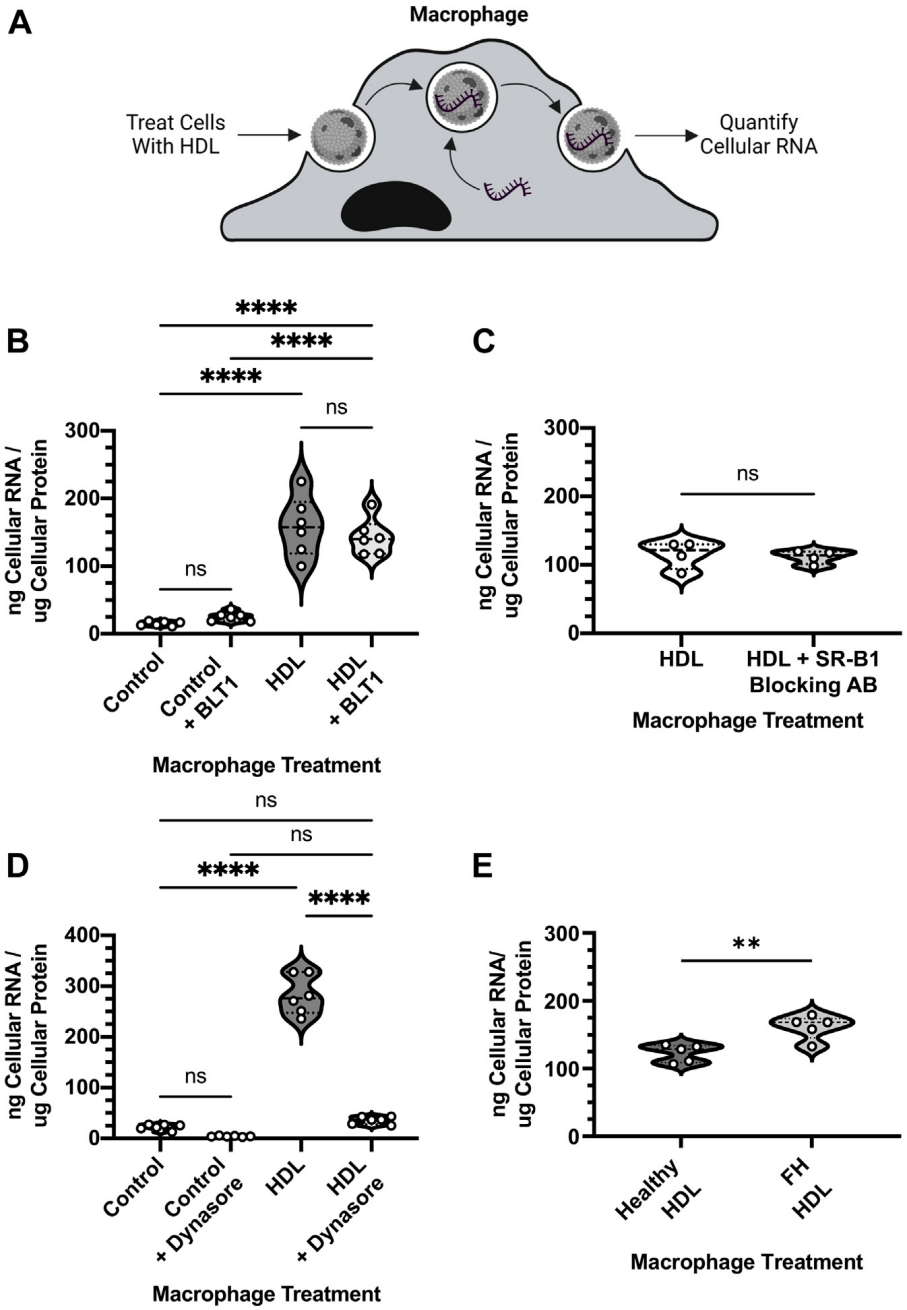
HDL induced macrophage RNA export. A: Diagram of experiment. Media isolated from control and HDL-supplemented J774 macrophages, including BLT1 (B), SR-BI blocking antibodies (C), and Dynasore (D) treated cells, were analyzed by fluorometry to quantify cellularly-derived RNA content (n = 3 per group; technical duplicates). E: Media isolated from BMDMs treated with either HDL from healthy or familial hypercholesterolemia patients were analyzed by fluorometry to quantify cellularly-derived RNA content (n=5 per group; technical duplicates). Values are reported as violin plots showing median, quartile ranges, and all individual values (B, C, D, and E). One-way ANOVA (Tukey test) (B, C, D) and Student’s *t-*test (E) results were as follows: ns *P* ≥ 0.05, ∗∗ *P* < 0.01, or ∗∗∗∗ *P* < 0.0001. BLT-1, block lipid transport-1; BMDM, bone marrow-derived macrophage; SR-BI, scavenger receptor, class B type I.

To determine if HDL have the capacity to accept cellular RNA export from macrophages, J774 cells were labeled with SYTO dye prior to HDL incubation with macrophages (Fig. 4A). Little to no SYTO stained RNA were released from labeled macrophages in the absence of HDL in the media, suggesting that SYTO labeled RNAs are not loaded into EV released from J774 macrophages, or the level of released EVs are too small to detect (Fig. 4B). The cellular efflux of RNA was significantly increased (*P* < 0.0001) when HDL was present in the accepting media, as 158.30 ng of cellular RNA / μg cellular protein were quantified in the HDL-containing media (Fig. 4B). To ensure that unbound SYTO dye is not being released from the cell into the media, media from untreated and SYTO-treated cells were collected and purified RNA were added to the collected media to quantify a potential increase in SYTO fluorescence. There were no significant increases in SYTO fluorescence signal between the RNA supplemented media and nonsupplemented media taken from control cells or those labeled with SYTO (supplemental Fig. S4), showing that there was no quantifiable accumulation of free SYTO dye that has escaped the cells. SR-BI is a transmembrane receptor protein that facilitates the HDL-mediated bidirectional flux of lipids and has been shown to promote retro-endocytosis of HDL upon holo-particle uptake (18). BLT-1 is a small molecule inhibitor of SR-BI-mediated exchange of lipids with HDL (19). BLT-1 also increases the binding affinity and decreases the dissociation rate of HDL to SR-BI (20). While BLT-1 inhibits lipid transfer, it does not interfere with endocytic pathways, secretory pathways, and does not impact cytoskeletal networks within the cell (20). To determine if HDL-mediated flux of cellular RNA is dependent on SR-BI-mediated lipid efflux, we quantified RNA export from macrophages treated with BLT-1. Results from these studies did not show significant differences in HDL-mediated export of RNA from macrophages treated with BLT-1 compared to control nonHDL-mediated export (Fig. 4B). Additionally, HDL-mediated flux of cellular RNA from macrophages was not significantly changed by inhibiting HDL binding to SR-BI using blocking antibodies in vitro (Fig. 4C). To determine if macrophage endo-lysosomal processing regulates macrophage RNA export to accepting HDL, macrophages were treated with the small molecule inhibitor Dynasore. Dynasore has been shown to inhibit clathrin-mediated endocytosis and the progression of the endo-lysosomal system by modulating V-ATPase activity (21). Strikingly, Dynasore treatments resulted in a significant 87.4% decrease (*P* < 0.0001) in RNA export to HDL in macrophages (Fig. 4D). These data suggest that endo-lysosomal maturation and functional integrity is critical to macrophage RNA export to HDL, possibly through HDL holo-particle uptake and retro-endocytosis, in a pathway independent of SR-BI. Previous studies have shown that apoE mediates HDL binding and cholesterol efflux through interactions between its N-terminal structural motifs (22). Patients with familial hypercholesterolemia (FH) have increased levels of HDL-associated apoE which is believed to play a role in aberrant HDL catabolism (23, 24). Thus, we hypothesized that HDL from FH patients may have an increased ability to mediate the export of RNA from macrophages by modifying the binding affinity of HDL in an apoE-mediated mechanism. To test this, we labeled BMDMs with SYTO prior to treatment with healthy or FH HDL and analyzed their RNA export capacity (Fig. 4E). HDL derived from healthy patients exhibited a cellular RNA export capacity of 122.80 ng of cellular RNA / μg cellular protein (Fig. 4E). The cellular RNA export capacity was significantly increased (*P* < 0.01) when cells were given HDL derived from FH patients as an acceptor of RNA export, as 161.30 ng of cellular RNA / μg cellular protein was quantified in the HDL-containing media (Fig. 4E). These data show that HDL derived from FH patients have a greater capacity to induce cellular RNA export from macrophages compared to healthy HDL, possibly by modifying the binding interactions of HDL to macrophages (24). While FH HDL exhibited an increased capacity to accept RNA from macrophages, quantification of total RNA carried by FH HDL showed no significant differences when compared to healthy control HDL (supplemental Fig. S5A). Quantification of total HDL cholesterol showed a significant decrease in cholesterol-content of FH HDL in comparison to healthy control HDL (supplemental Fig. S5B). Together these data indicate that the endo-lysosomal system plays a critical role in HDL-mediated export of cellular RNAs, that the HDL-mediated pathway of cellular RNA acceptance is likely independent of SR-BI, and that the underlying processes are impacted by disease-associated changes to HDL, specifically in the context of FH.

### HDL delivery of extracellular RNA to macrophages

**Fig. 5:**
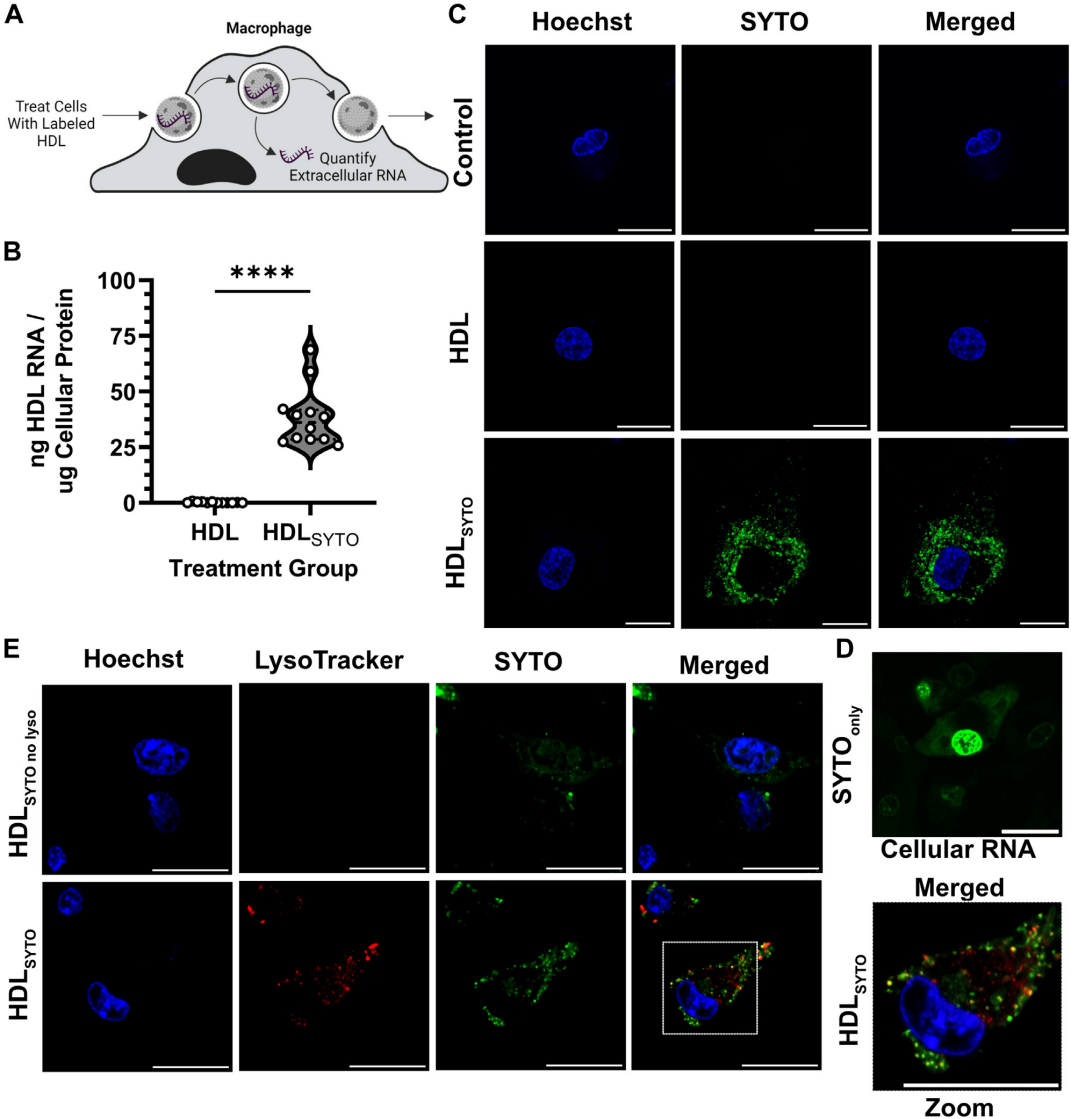
HDL delivery of RNA to macrophages. HDL-derived RNA fluorescence was analyzed in macrophages in vitro. A: Diagram of experiment. B: HDL-derived RNA uptake was quantified utilizing fluorometry and normalized to total cellular protein (n = 3 independent lipoprotein samples; technical quadruplets). C: Confocal microscopy of live cells in control, HDL-treated, and SYTO-labeled HDL treated groups to image HDL mediated delivery of labeled RNA (Scale bar =20 nm). D: Confocal microscopy of live cells stained with SYTO to image the cellular distribution of RNA in untreated cells. E: Confocal microscopy of live cells treated with SYTO-labeled HDL, with and without lysosomal dye, was performed to characterize the subcellular localization of HDL-derived RNA within the endo-lysosomal system. (Nuclear stain – Blue, Lysosomal stain – Red, RNA stain – Green) (Scale bar = 20 nm). Values are reported as violin plots showing median, quartile ranges, and all individual values (B). Student’s *t-*test (B) results were as follows: ns *P* ≥ 0.05 or ∗∗∗∗ *P* < 0.0001.

To determine if HDL has the capacity to deliver extracellular RNA to macrophages, cells were treated with SYTO-stained HDL for 24h. To quantify HDL-RNA delivery, recipient macrophages were washed and intracellular fluorescence was measured (Fig. 5A), which showed that J774 macrophages took up 38.48 ng of HDL-RNA / μg of cellular protein (Fig. 5B). Live cell imaging showed that HDL-RNA were taken up by macrophages, as indicated by the presence of punctate fluorescence (SYTO) signal in cells treated with labeled HDL and not in those treated with the vehicle alone or nonlabeled HDL (Fig. 5C). The patterns of HDL delivered RNA were observed in punctate signals in non-nuclear macrophage organelles (Fig. 5C). The HDL-delivered RNA pattern of SYTO signal is dramatically different than SYTO staining of cellular RNA in macrophages without HDL delivery, which was predominantly found in the nucleus (Fig. 5D). We hypothesized that HDL-derived RNA would be trafficked through the endo-lysosomal pathway; thus, we utilized a pH sensitive live cell dye (Lysotracker Deep Red, Invitrogen) to assess colocalization of HDL-delivered RNA within the acidic environment of the lysosome (Fig. 5E). Strikingly, HDL-delivered RNA was detected in lysosomes, as indicated by colocalization of SYTO and Lysotracker dyes. These results support that that HDL-delivered RNA is trafficked through the endo-lysosomal system.

### Dendritic cell to macrophage HDL-sRNA intercellular communication

**Fig. 6:**
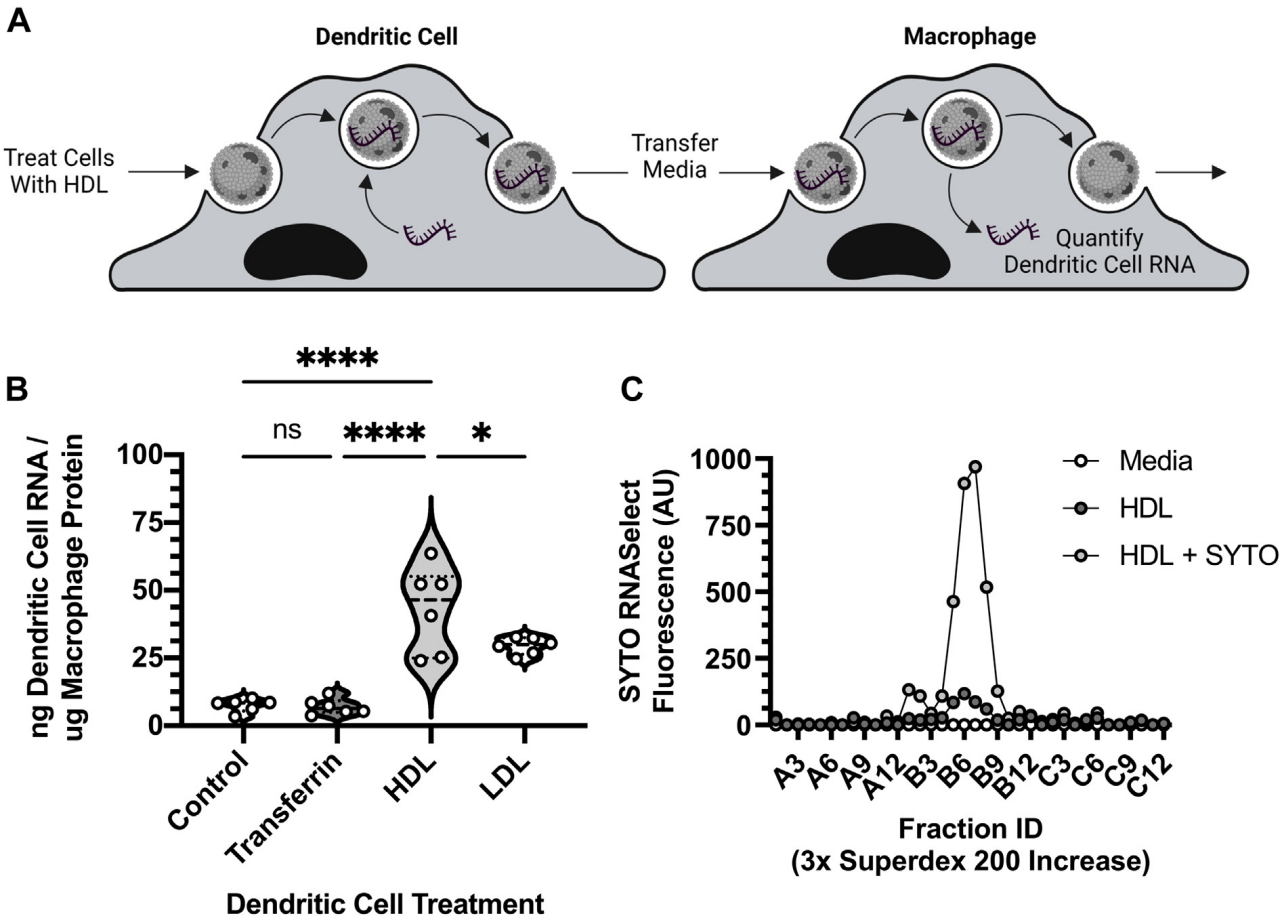
HDL-mediated intercellular communication between immune cells. Following sequential incubations with supplemented media (i.e., transferrin, HDL, and LDL) in vitro, BMDC-derived RNA was quantified in BMDM cells A) Diagram of experiment. B: BMDMs were analyzed by fluorometry to quantify BMDC-derived RNA content (n = 3 monocyte derived cell groups; technical quadruplicates). C: Following the treatment of BMDCs with HDL, media samples were purified utilizing size-exclusion chromatography and SYTO signal was analyzed by fluorometry (n = 1 per group). Values are reported as violin plots showing median, quartile ranges, and all individual values (B). One-way ANOVA (Tukey test) (B) results were as follows: ns *P* ≥ 0.05 or ∗∗∗∗ *P* < 0.0001. BMDM, bone marrow-derived macrophage; BMDC, bone marrow-derived dentric cell.

As previous experiments showed that HDL can deliver extracellular sRNAs to recipient cells and mediate cellular RNA export from macrophages in vitro, we hypothesized that HDL may have the capacity to transport RNA between immune cells as a mediator of intercellular communication. To test this hypothesis, bone marrow cells were isolated from mice and differentiated into either BMDMs or BMDCs. To label donor cell RNA, BMDCs were stained with STYO dye and washed prior to incubation with HDL-supplemented media, LDL-supplemented media, transferrin-supplemented media or nonsupplemented control media. Following these incubations, conditioned media was collected and transferred to recipient BMDMs (Fig. 6A). Fluorescence analyses of recipient BMDMs receiving HDL-supplemented media found that recipient macrophages received 42.99 ng of BMDC RNA / μg of BMDM cellular protein by HDL transfer (Fig. 6B). A negative SYTO-only control condition was tested consisting of BMDCs that were labeled with SYTO, but did not receive HDL as an acceptor of cellular RNA export. BMDMs treated with control media received very little RNA by nonHDL transfer 7.55 ng of BMDC RNA / μg of BMDM cellular protein (Fig. 6B). We selected transferrin to serve as a negative protein control for this assay because it has not been shown to be an RNA binding protein. Fluorescence analyses of recipient BMDMs receiving transferrin-supplemented media found that recipient macrophages received 7.02 ng of BMDC RNA / μg of BMDM cellular protein; a value that is not statistically different from cells treated from the SYTO-only control (Fig. 6B). Most interestingly, analyses showed that BMDMs receiving LDL-supplemented media showed that recipient macrophages received 29.42 ng of BMDC RNA / μg of BMDM cellular protein; a value is statistically lower than those observed in HDL-supplemented condition (*P* < 0.05) but statistically increased compared to both the SYTO-only (*P* < 0.001) and transferrin-supplemented (*P* < 0.001) conditions (Fig. 6B). To confirm that HDL acceptors in conditioned media bound SYTO-stained BMDC cellular RNA, media samples were analyzed by SEC and fluorometry (Fig. 6C). Consistent with the data from the BMDM uptake experiment, results indicate that there was an increase in BMDC-derived cellular RNA present on accepting HDL. These data show that HDL can mediate the transfer of BMDC RNA to BMDM cells and plays a role in intracellular RNA communication between immune cell types.

## DISCUSSION

This study provides critical new information to the field of lipoprotein-mediated transport of sRNAs regarding the quantity of extracellular sRNA being trafficked by lipoproteins in plasma and the quantification of HDL capacity to serve as both an acceptor of sRNAs from donor cells and a deliverer of RNAs to recipient cells. Results support that HDL have the ability to transfer cellular RNA from dendritic cells to macrophages. These results support a novel cell-to-cell communication pathway between disparate immune cell types. These observations were made possible through the use of SYTO RNASelect dye, which allowed for the absolute quantification of sRNAs on HDL without the need for RNA isolation. Moreover, SYTO dye facilitated the tracking and quantification of RNA transfer between immune cells.

HDL, among other lipoprotein classes, have been the target of many studies attempting to uncover its roles in extracellular RNA transport since being described just over a decade ago (6, 7, 8, 25). Technologies developed in this new field have accelerated rapidly and have allowed for significant progress to be made in the identification of a myriad of host and nonhost RNAs found on these lipoproteins, as well as defining the roles that these diverse molecules play in the context of disease (9, 11, 26). Despite the rapid progress that has been made in the field, current techniques require isolating RNA, preparing cDNA by reverse transcription, and analyzing these transcripts through candidate-based or noncandidate-based approaches (e.g., PCR, sRNA sequencing) (10). Although the SYTO approach used in the study does not provide the identification or post-transcriptional modifications of RNAs being transported by lipoproteins as other techniques do, it provides critical insights regarding the ability of HDL to mediate the transport of RNA cargo between cells. Most importantly, SYTO allows for the absolute quantification of RNA cargo on circulating lipoproteins and intercellular communication without the need for RNA isolation which introduces many biases. This study provides new insights to the field of extracellular RNA transport in RNA-based intercellular communication mediated by lipoproteins, EVs, and RNA-binding proteins. Although EVs have been reported to transfer RNA between cells, many other carriers do as well, including lipoproteins, natural nanoparticles (e.g., supermeres), viral antigen particles, RNPs, and many others. HDL transport and delivery of extracellular sRNAs is not likely an alternative mechanism to EV transfer of RNA. HDL and EV-sRNA biology likely have their own distinct mechanisms, receptors, processes, cargo, and biophysical properties. The SYTO approach could enable researchers to identify key regulators of cellular RNA transfer to and from RNA-binding proteins utilizing numerous methods (e.g., chemical inhibition, gene silencing, and genetic knockout) that would allow for the variance in cellular export or uptake to be determined. As observed in our BLT-1 treatments of macrophages, our data suggest that HDL-mediated RNA export is independent of SR-BI mediated transfer of lipids to HDL and that increasing HDL binding affinity to SR-BI does not increase RNA export. Likewise, inhibition of HDL binding to SR-BI using blocking antibodies did not impact RNA export from macrophages in vitro. These data suggest that the HDL-mediated pathway of RNA acceptance from macrophages is likely independent of SR-BI function. Utilizing Dynasore as an inhibitor of endo-lysosomal progression, our data suggests that progression of HDL through the endo-lysosomal system is critical to HDL-mediated RNA export. These data may suggest that HDL-mediated RNA export follows a retro-endocytosis pathway that has previously been suggested for cholesterol efflux (18, 27, 28, 29).

Previous studies have highlighted the link between HDL composition and function (i.e., cholesterol efflux) (4, 23, 24, 30, 31). Previous studies have shown that HDL metabolism is altered in patients with FH and these observed changes in HDL function are likely driven by changes in the particle composition (24). As observed in our RNA export experiments, HDL isolated from patients with FH had an increased capacity to induce cellular RNA export from macrophages. Our findings may partially explain results from previous studies which indicate that HDL from FH patients contains greater concentrations of total miRNAs (32). Patients with FH have a notable increase in HDL-associated apoE which has been shown to mediate HDL metabolism by altering its interaction with membrane receptors (22, 24). These proteomic changes observed in FH may be responsible for the observed differences in RNA export capacity by altering the endocytosis of HDL into macrophages through a receptor mediated process; however, this model requires further investigation. Moreover, it is unclear what nonproteomic compositional changes of HDL in patients with FH may contribute to their ability to accept RNA from macrophages (i.e., reduced HDL-C, TG enrichment). Although quantification assays of total HDL-associated RNA showed no significant differences between HDL from healthy or FH patients, total cholesterol levels were significantly decreased in FH HDL compared to healthy HDL. Such compositional differences in the cholesterol content of HDL may contribute to the increased capacity of FH HDL to accept RNAs from cells; however, additional structure-function-cargo analyses will be required in future studies.

Results from SYTO analyses suggest that both HDL and LDL transport μg quantities of RNA per mg of total protein. This level of RNA cargo has the potential to activate RNA-sensing toll-like receptors (TLR) within the innate immune system if delivered to specific immune cells. As shown by live cell imaging, our data supports the hypothesis that HDL-delivered RNAs are taken up into the endo-lysosomal compartment of recipient macrophages where they could play a role in TLR signaling. The approaches outlined in this study would enable future studies to characterize the pathway of HDL or LDL-delivered sRNAs through the endo-lysosomal system, characterize the modulators of this process, and colocalize these RNA cargos with TLRs that drive inflammatory pathways. As indicated by previous reports of proinflammatory TLR signaling derived from LDL-associated RNA, it may be possible that HDL’s anti-inflammatory role is mediated, in part, by inducing the efflux of LDL deposited proinflammatory sRNAs within the endo-lysosomal system (33). Historically, it has been shown that HDL plays anti-inflammatory roles in macrophages by its capacity to mediate the efflux lipids from burdened cells; however, HDL can become proinflammatory in disease and HDL’s ability to transfer extracellular sRNAs to recipient macrophage endosomes may contribute to HDL functionality in disease. We posit that HDL delivers its sRNA cargo indiscriminately to cells through either lipoprotein selective core uptake of HDL’s cargo or by holo-particle uptake by receptors and/or phagocytosis. Once inside the cytoplasm or endo-lysosome compartment, HDL-sRNAs likely regulate gene expression and cellular phenotypes through both singular and collective actions.

Previous studies have characterized the pathways involved in HDL-mediated export of host-derived miRNAs from beta cells; however, the pathways by which lipoproteins obtain nonhost sRNAs are poorly understood (34). The characterization of these pathways could be aided using SYTO approaches outlined in this study. For example, HDL and LDL are highly-enriched with microbial sRNAs (9). Cellular RNA of microbial species (e.g., bacteria, fungi) could be labeled with SYTO prior to transplanting these microbes into the microbiome or specific tissues (e.g., mucosa of the gastrointestinal tract, respiratory system). From these sites, we could evaluate the source and mechanisms of microbially-derived RNAs that may be trafficked to phagocytotic cells within these tissues which may serve as an interface for lipoprotein acquisition.

Although the approaches outlined in this paper offer clear advantages in characterizing and quantifying lipoprotein-mediated RNA transport over conventional tools, it has its own limitations and should be utilized with orthogonal methods that offer sequence identification and epitranscriptomic analysis of the RNA cargo. For example, the use of SYTO RNA labeling approaches provides researchers with a powerful tool to assess global sRNA flux related to lipoproteins that can be followed by sequencing or PCR studies to evaluate the individual identification, cellular origin, and potential biological functions of candidate sRNAs. To summarize, this study a) provides isolation-free wholistic quantification of lipoprotein-associated RNA cargos, b) characterizes the ability of HDL to accept and deliver RNA cargos to cells, c) demonstrates the impact of disease (FH) on HDL-mediated RNA efflux, and d) defines the ability of HDL to serve as a mediator of cellular RNA transfer between immune cells. This study indicates that the sheer mass of RNA cargos carried by lipoproteins, accepted from cells, and delivered to recipient cells by HDL has been unappreciated utilizing previously described methods and offers critical insights to the field of extracellular RNA transport.

## Data availability

Data that support the findings of this study are available from the corresponding author, KCV, upon reasonable request.

## Supplemental data

This article contains supplemental data.

## Conflict of interest

No author reports a financial interest with respect to the work outlined in this document.
