# Supplementary material for: High-density lipoproteins mediate small RNA intercellular communication between dendritic cells and macrophages
Source: J Lipid Res. 2023 Jan 7;64(2):100328. doi: 10.1016/j.jlr.2023.100328 (PMC9929858; doi:10.1016/j.jlr.2023.100328)
Supplement: Supplementary figures [file mmc1.docx]

**
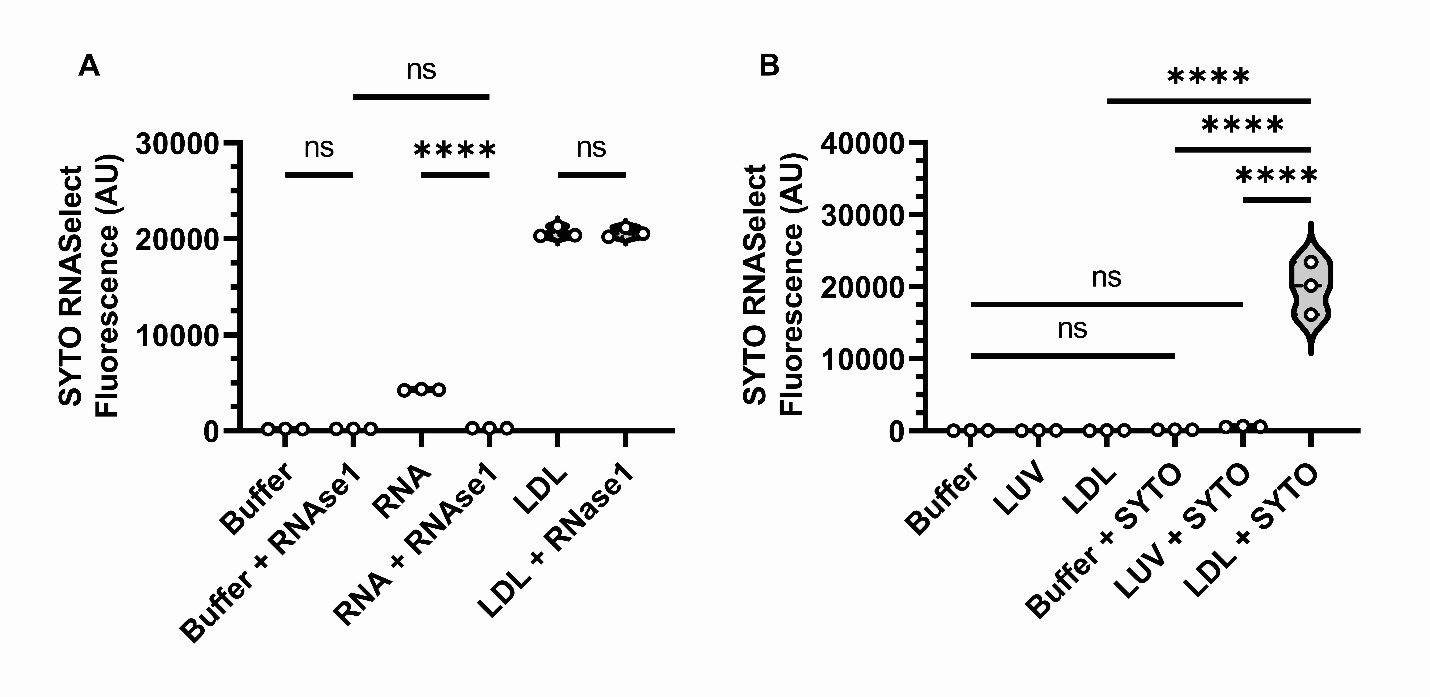
**

**Supplemental Figure 1. Quantification and characterization of LDL RNA utilizing fluorescence.** Fluorescence was quantified in samples containing purified RNA (**A**) and LDL (**A, B**) and sensitivity of RNA degradation was observed by treating samples with RNase1 (**A**). The RNA fluorescence of independent LDL (**B**) samples was compared to buffer and large unilamellar vesicles (LUV). Values are reported as violin plots showing median, quartile ranges, and all individual values (**A**, **B**). One-way ANOVA (Tukey test) (**A**, **B**) results were as follows: ns ≥0.05 or **** P<0.0001.


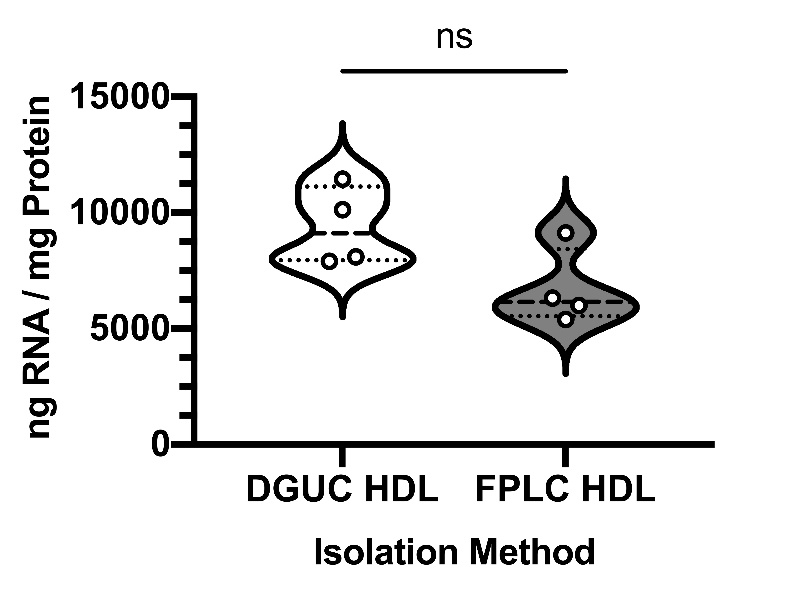


**Supplemental Figure 2. Comparison of total HDL RNA from lipoproteins purified using ultracentrifugation and FPLC purification.** Total RNA content of HDL were quantified using SYTO Green RNASelect from HDL samples purified by either DGUC or FPLC. FPLC purified HDL samples were purified by injecting plasma onto size-exclusion chromatography columns, pooling eluant fractions containing HDL, and calculating lipid-associated RNA within these fractions using Cleanascite lipid removal reagent. Values are reported as violin plots showing median, quartile ranges, and all individual values. Student’s *t*-test results were as follows: ns P≥0.05.

**
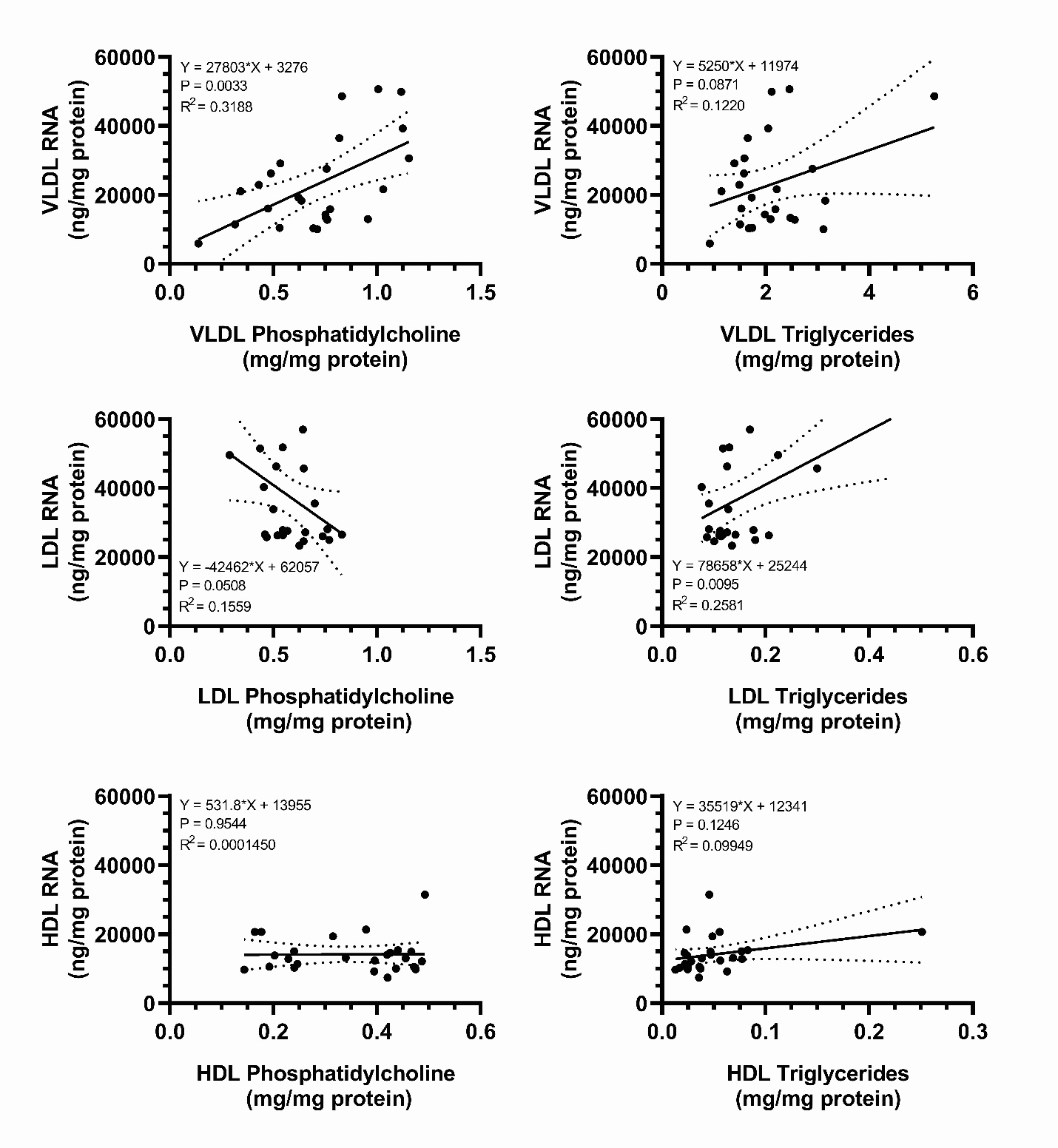
**

**Supplemental Figure 3. Correlations between the primary lipid components and RNA in DGUC Lipoproteins.** Total phosphatidylcholine and triglyceride concentrations were quantified by colorimetric analyses and correlated to their RNA cargo in VLDL, LDL, and HDL. (n=25 per group). Values are shown as linear regression plots with all individual values. Regression results were plotted with the best line of fit and 95% confidence bands.


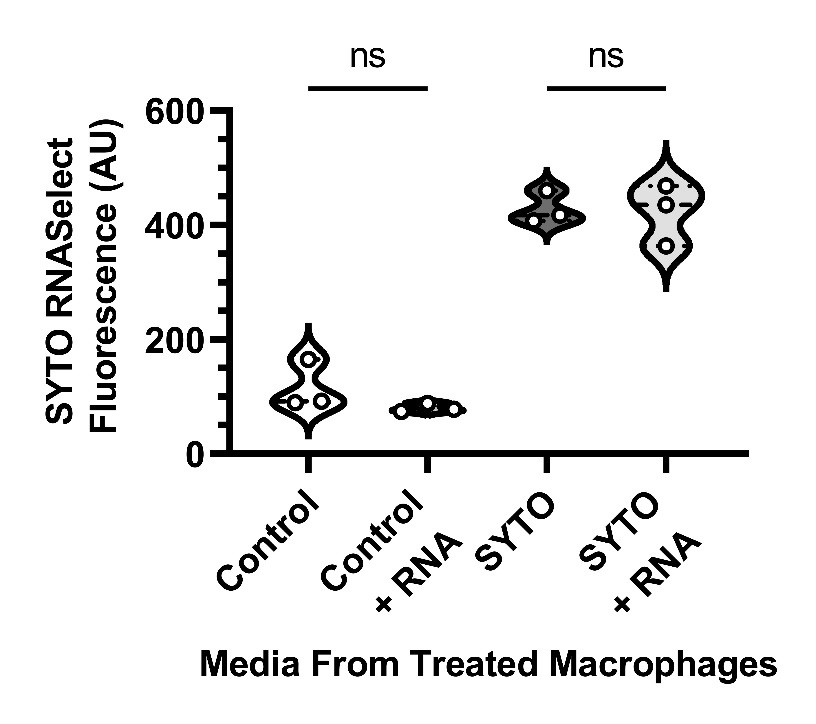


**Supplemental Figure 4. Accessing SYTO stability of treated macrophages *in vitro*.** Media from untreated and SYTO labeled cells were analyzed by fluorimetry after the addition of buffer or purified RNA to evaluate unbound SYTO in the media (n=3).


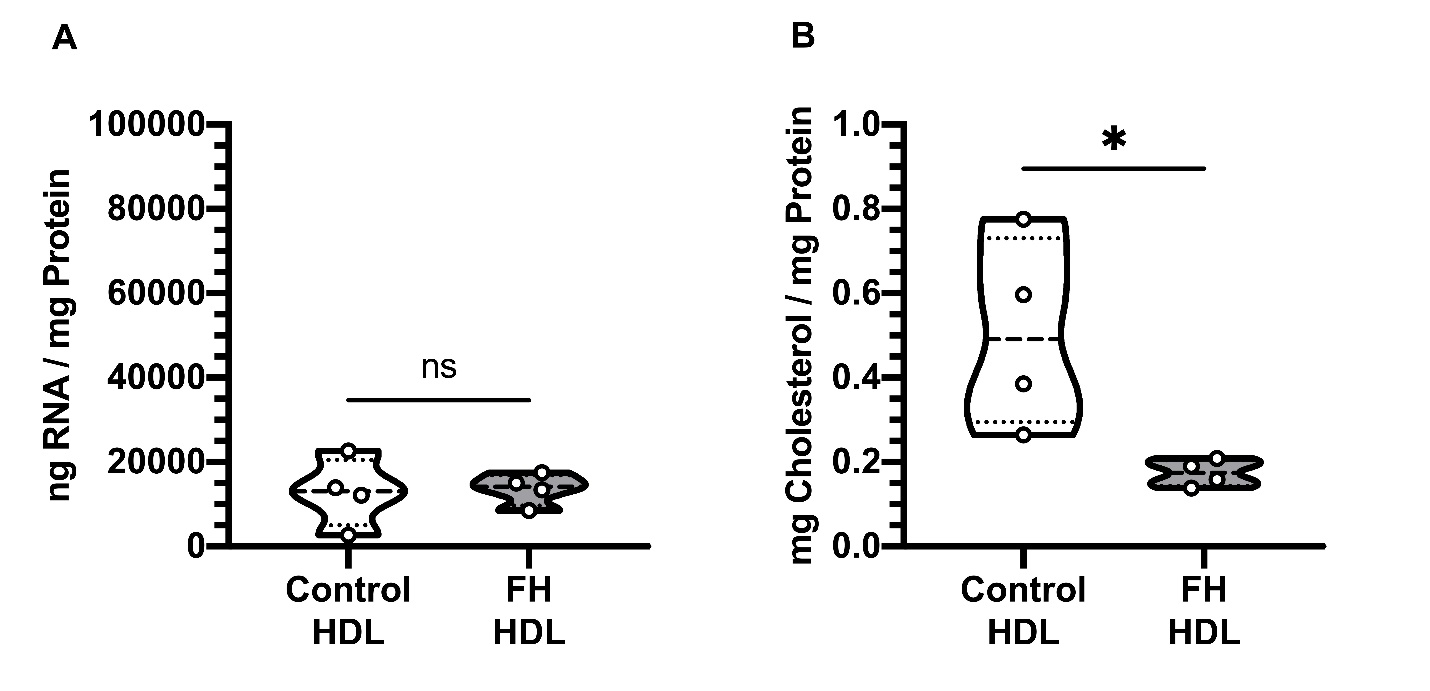


**Supplemental Figure 5. Comparison of HDL-associated RNA and cholesterol cargo from healthy and familial hypercholesterolemia patients.** The RNA (**A**) and cholesterol (**B**) cargo of DGUC HDL samples from healthy and familial hypercholesterolemia patients were quantified and normalized to total protein of each sample (n=4 per group). Student’s *t*-test (**A**, **B**) results were as follows: ns P≥0.05 or * P<0.05.
